# Effects of Dietary β-Mannanase Supplementation on Growth Performance, Apparent Total Tract Digestibility, Intestinal Integrity, and Immune Responses in Weaning Pigs

**DOI:** 10.3390/ani10040703

**Published:** 2020-04-17

**Authors:** Jae-Cheol Jang, Kwang Ho Kim, Young Dal Jang, Yoo Yong Kim

**Affiliations:** 1 Department of Agricultural Biotechnology, and Research Institute of Agriculture and Life Sciences, Seoul National University, Seoul 08826, Korea; jchang0720@hanmail.net (J.-C.J.); kkh830625@naver.com (K.H.K.); youngdal.jang@uwrf.edu (Y.D.J.); 2 Department of Animal and Food Science, University of Wisconsin-River Falls, River Falls, WI 54022, USA

**Keywords:** acute phase protein, β-mannanase, growth performance, intestinal morphology, oxidative stress, apparent total tract digestibility, weaning pigs

## Abstract

**Simple Summary:**

Many plant-based feedstuffs contain non-digestible factors, such as β-mannans, that may reduce growth performance, as well as energy and nutrient digestibility. However, weaning pigs lack enzymes—such as β-mannanase—necessary to more completely digest β-mannan. Therefore, this experiment aimed to investigate the effects of dietary β-mannanase supplementation on growth performance, apparent total tract digestibility of nutrients, intestinal integrity and the immunological and oxidative stress parameters of weaning pigs. Our result suggested that adding β-mannanase to the weaning pigs’ diet increased the apparent total tract digestibility (ATTD) of ether extract, jejunum villus height, and villus height-to-crypt depth ratio, and lowered crypt depth compared with those fed the no-β-mannanase diet. This study indicated that the inclusion of β-mannanase showed the potential to improve fat digestibility, intestinal development and gut health of weaning pigs.

**Abstract:**

The experiment aimed to investigate the effects of dietary β-mannanase supplementation on growth performance, apparent total tract digestibility (ATTD) of nutrients, intestinal integrity, and the immunological and oxidative stress parameters in weaning pigs. A total of 64 newly weaning pigs (initial body weight: 6.96 ± 0.70 kg) were allotted to two dietary treatments in eight replicates per treatment with four pigs per pen based on body weight and sex. Dietary treatments were 1.) CON (control: corn-soybean meal based basal diet) and 2.) β-mannanase (basal diet +0.06% β-mannanase). The β-mannanase supplementation did not affect growth performance, concentrations of acute phase protein, superoxide dismutase and glutathione peroxidase. However, the pigs fed the β-mannanase-supplemented diet had greater ATTD of ether extract, jejunum villus height, and villus height-to-crypt depth ratio, and lower crypt depth compared with those fed the CON diet (*p* < 0.05). The pigs fed the β-mannanase-supplemented diet tended to have the lower count of *E. coli* in cecum than those fed the CON diet (*p* = 0.08). In conclusion, dietary β-mannanase supplementation did not affect growth performance, immune response and oxidative stress of weaning pigs, whereas it increased fat digestibility and had positive effects on intestinal integrity and cecum microflora by reducing the count of *E.coli*.

## 1. Introduction

Swine diets normally contain certain amounts of non-starch polysaccharides (NSP) that could negatively influence digestive physiology and gut dynamics [1]. As one group of NSP, β-mannan is a part of hemicellulosic polysaccharide, and the second most abundant in nature [2]. The content of β-mannan in soybean meal (SBM) is 1.3% to 1.5% [3], 0.4% in barley, and less than 0.1% in corn [4]. In pigs, the β-mannan is poorly digested in the digestive tract due to lack of endogenous enzymes targeting α-1,6-galactosyl and β-1-4-mannosyl bonds of it [5]. Although β-mannan content in those ingredients is relatively low, it could negatively influence nutrient digestion and increase intestinal viscosity, resulting in adverse effects on immune system and gut microflora of weaning pigs [6,7]. Therefore, adding an exogenous β-mannanase to the weaning diet could be a viable option to mitigate these negative impacts of β-mannan for weaning pigs.

The β-mannanase is one of the endo-carbohydrases, playing a role in degrading β-mannan to manno-oligosaccharide (MOS) and mannose [8]. Benefits from the β-mannanase supplementation in the corn-SBM diet on the growth performance and nutrient digestibility of nursery pigs have been reported [9,10,11]. However, the effects of β-mannanase supplementation to the weaning pigs’ diet are still inconsistent and there is limited information available about how the β-mannanase supplementation influences innate immunity and microbial proliferation in the gut of weaning pigs.

Therefore, the objective of the present experiment was to demonstrate the effect of dietary β-mannanase supplementation on growth performance, apparent total tract digestibility (ATTD), intestinal integrity, immune response, and oxidative stress of weaning pigs.

## 2. Materials and Methods

All experimental procedures involving animals were conducted in accordance with the Animal Experimental Guidelines provided by the Seoul National University Institutional Animal Care and Use Committee (SNU-200401-4).

### 2.1. Animals, Diets, Housing, and Feeding

A total of 64 weaned pigs ((Landrace × Yorkshire) × Duroc) with an average initial body weight (BW) of 6.96 ± 0.70 kg and a weaning age of 28 ± 3 days were allotted to two treatments in eight replicates with four pigs (two barrows and two gilts) per pen based on BW and sex. Dietary treatments were 1) control (CON; corn-SBM-barley based basal diet) and 2) β-mannanase (basal diet +0.06% β-mannanase; 96,000 U/kg diet). The β-mannanase (Hemicell-HT^®^, a minimum of 160 million unit mannanase per kg product, Elanco Animal Health, Greenfield, IN, USA) was added to the basal diet (CON) by replacing corn. Experimental diets (Table 1) were prepared in mash form over the two phases (Phase 1: day 0 to 14 post-weaning; Phase 2: day 14 to 35 post-weaning). The Phase 1 diet was formulated to contain 3400 kcal/kg metabolizable energy (ME), 1.35% standardized ileal digestibility (SID) lysine, 0.80% total calcium and 0.40% standardized total tract digestibility (STTD) phosphorus, and the Phase 2 diet was formulated to contain 3350 kcal/kg ME, 1.23% SID lysine, 0.70% total calcium and 0.40% STTD phosphorus. All diets were formulated to meet or exceeded the nutrient requirements as recommended by National Research Council (NRC [12]).

The piglets were housed in partially slatted concrete floor pens (0.90 × 2.15 m^2^). Each pen was equipped with a self-feeder and low-pressure nipple drinker to allow ad libitum access to feed and water.

### 2.2. Experimental Procedures and Measurement

The pigs were individually weighed at the start of the trial, day 14, and day 35 post-weaning. The pen-based feed disappearance was measured when the pigs were weighed and average daily gain (ADG), average daily feed intake (ADFI) and gain-to-feed (G:F) ratio were calculated.

At day 0 (initial), 14 and 35 post-weaning, blood samples (10 mL) from five pigs selected based on the average BW in each treatment were collected via jugular venipuncture in disposable vacutainer tubes (Becton Dickinson, Franklin, NJ, USA). After centrifugation (3000× *g* for 15 min at 4 °C), serum samples were separated and stored at −20 °C and later analyzed for α-1-acid glycoproteins (AGP) and haptoglobin (HP) as the parameters for acute phase protein (APP), and for superoxide dismutase (SOD) and glutathione peroxidase (GPx) as the parameters for oxidative stress.

After the termination of the feeding trial, a total of ten pigs (five pigs per treatment, average BW: 17.17 ± 0.47 kg) were selected based on the average BW and slaughtered after 12 h fasting. Digesta samples were collected from ileum (about 100 cm before the ileo-cecal ostium), cecum and colon (about 50 cm beyond the cecal–colon junction). The digesta collected from all sampling sites was transferred immediately to sterile plastic tubes and subsamples were taken for bacterial enumeration.

The digestive tract was removed and sections of jejunum (5.5 m from stomach sphincter) and ileum (10 cm prior to the ileo-cecal orifice) were excised, opened along their length at the mesenteric border, fixed in 10% formaldehyde solution, and embedded in paraffin wax. These tissue samples were cut in a transverse section in 5 mm thick slices and stained with haematoxylin and eosin. Villus height (VH), crypt depth (CD), and villus height-and-crypt-depth ratio (VCR) were measured at 10× magnification using an Olympus BX61 microscope and image analysis software (analySIS Pro, Olympus Belgium, Aartselaar, Belgium).

For the ATTD and nitrogen retention, a total of 10 barrow piglets (initial average BW 10.17 ± 1.35 kg) were selected separately from the feeding trial and allocated to each treatment in five replicates in a completely randomized design (CRD). They were housed individually in the metabolic cage for the adaptation and collection periods. During the adaptation and collection periods, water was provided ad libitum. The experimental diet was provided twice a day at 07:00 and 19:00 h by three times the maintenance energy requirement (106 kcal of ME/kg of BW^0.75^; NRC, [15]). There was an initial seven day adaptation period followed by the collection period on which all feces and urine were collected. The first and final meals in the collection period contained 0.5% chromium oxide and ferric oxide, respectively, as an indigestible indicator. Excreta collection was started when the initial marker was observed in the feces and terminated when the last marker appeared in the feces, following the methods described by Adeola [16]. The feces and urine collected from each pig for five days was stored at −20 °C until further analysis. After the collection period, the fecal samples were dried in a forced air-drying oven at 60 °C for 72 h, and ground in a Wiley mill using a 1 mm screen and analyzed for dry matter (DM), crude protein (CP), ether extract (EE), and ash for the ATTD calculation.

### 2.3. Chemical Analyses

Diet and fecal samples were analyzed based on the AOAC [17] analytical methods; DM (method 930.15), CP (method 990.03), EE (method 920.39), and ash (method 942.05).

Analyses of SOD and GPx in serum were measured using commercially available assay kits (Cayman Chemical Company; Ann Arbor MI, USA; catalog number: SOD = 706,002 and GPx = 703,102) according to the recommendations from the manufacturer, with assays run in triplicate in 96-well microplates and an intra-assay CV of ≤ 10.0%.

The sera were also analyzed for AGP and HP using commercial ELISA kits (EiAab^®^, Wuhan, China; catalog number: AGP = E0816p and HP = U0817p) according to the manufacturer’s instructions.

For the analysis of *E. coli* count, the digesta samples were diluted with sterile 0.9% NaCl solution in a 1:10 dilution. Then, it was further diluted from 10^−2^ to 10^−9^ for the *E. coli* count analysis. For the enumeration of the total number of *E. coli*, the suspensions were spread on Levine eosin-methylene blue (EMB) agar (Difco laboratories Inc., Detroit, MI, USA), and incubated under aerobic conditions at 37 °C for 24 h. Colonies with a green metallic sheen were counted as *E. coli*. The analysis was performed in duplicate for each sample and the result was reported by the average of the duplicate. The digesta microbial enumerations are expressed as log_10_ colony-forming units (CFU) in the fresh matter.

### 2.4. Statistical Analysis

All the data obtained in the current study were analyzed in accordance with a randomized complete block design using the GLIMMIX procedure of SAS (ver. 9.4. SAS Inst. Inc., Cary, NC, USA). A pen was used as an experimental unit for analysis of growth performance data. An individual pig was used as an experimental unit for the ATTD digestibility, intestinal morphology, blood analyses, and intestinal microflora. Least squares means were separated using the PDIFF option of SAS. The alpha level used for the determination of significance for all analysis was 0.05 and the tendency of all analyses was 0.05 < *p* < 0.11.

## 3. Results and Discussion

### 3.1. Growth Performance

In the current study, the β-mannanase did not affect ADG, ADFI, and G:F ratio over the entire phases (Table 2). The effects of β-mannanase supplementation on growth performance in weaning pigs is still inconsistent. Several studies reported the increased growth performance by β-mannanase supplementation in the weaning diet [9,18,19], mainly due to the energy sparing effect. Pettey et al. [9] reported that the β-mannanase supplementation in the weaning diet could increase the degradation of indigestible fractions and thus provide additional energy (approximately 100 kcal/kg of digestible energy (DE)] to weaning pigs. However, Huntley et al. [11] reported that supplementation of β-mannanase in the weaning diet did not provide additional DE, resulting in no effect on weaning pig growth. A possible reason for this inconsistent effect might be the various amount of β-mannan (one of soluble NSP; sNSP) and insoluble NSP (iNSP) in the diet. The iNSP are regarded as constituents of cell walls encapsulating nutrients, blocking the access of digestive enzymes to their substrates in the digestive tract [20]. The studies reported the increase in growth performance where the diets had less than 10% iNSP and 0.5% β-mannan [9,18], whereas the other study reported no effect of β-mannanase supplementation in growth performance where the diets had more than 10% iNSP and 0.5% β-mannan [11]. This indicated that the response of pigs to β-mannanase supplementation could depend on the content of β-mannan and iNSP in the diet. In the current study, no effects on growth performance by β-mannanase supplementation could be explained by a higher calculated content of iNSP and β-mannan in the diet (10.7% and 0.6%, respectively) compared with the studies that reported a positive effect on growth performance by β-mannanase supplementation [9,18].

In addition, the insufficient liberation of mannose by β-mannanase could be the other reason for the inconsistent effect of β-mannanase supplementation on growth performance. Kusakabe and Takahashi [21] reported that the sugar composition after hydrolysis of β-mannan by β-mannanase consisted of 3.3% mannose, 42% mannobiose, 20% mannotriose, 13.3% mannotetraose and 21.4% other oligosaccharides. This implies that a low amount of mannose is released from β-mannan by β-mannanase hydrolysis, and that β-mannanase supplementation might not show the energy sparing effect in the weaning diet, since mannose, which is only the energy-yielding fraction among mannan metabolites, was liberated by a relatively small amount by β-mannanase hydrolysis [22]. This supports the concept that the amount of absorbed simple sugars in the small intestine was insufficient to improve growth performance in the current study.

### 3.2. Apparent Total Tract Digestibility

The pigs fed the β-mannanase-supplemented diet had an increased ATTD of EE (*p* < 0.05) when compared to those fed the CON diet (Table 3), whereas the β-mannanase supplementation had no effects on the ATTD of DM, CP, ash, and nitrogen retention. The NSP content in the swine diet could depress lipid metabolism by the inhibition of lipolysis and intestinal fat absorption [23]. Especially, sNSP could impair the diffusion and connective transport of lipase, oil, and bile salt micelles within gastrointestinal contents, leading to change in the viscosity of digesta, and thus resulting in decreased fat digestibility [24]. Similarly, it was reported that in vitro lipolysis with gastric and pancreatic lipase was negatively affected by the emulsion prepared in the presence of high viscosity guar gum compared with that of low or medium viscosity [25]. Considering the β-mannans in SBM is mostly galactomannans, as one of the sNSP [3], which consist of water-soluble 1,4-linked β-D-mannopyranosyl residues [26], the increase in fat digestibility in this study might be attributed to the hydrolysis of β-mannan by β-mannanase resulting in reducing the amount of sNSP in the digesta.

### 3.3. Serum Oxidative Stress and Immune Parameters

**Figure 1:**
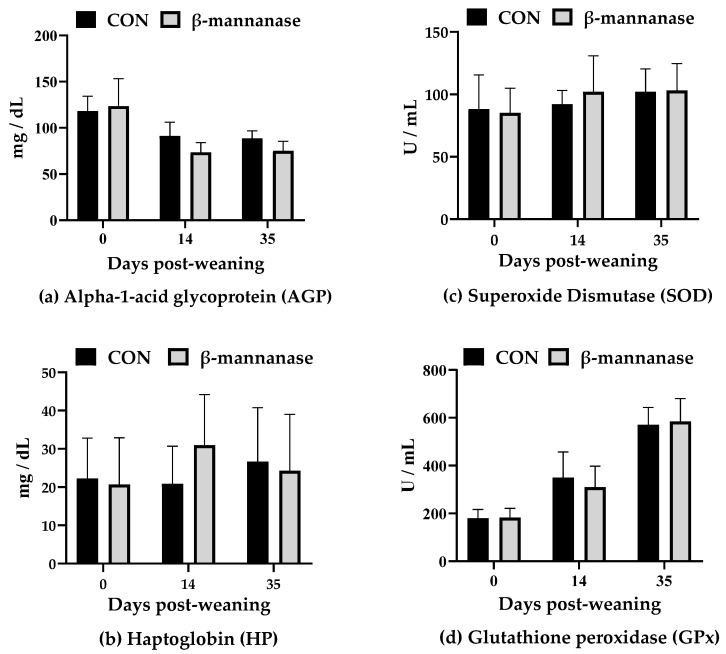
Effects of dietary supplementation of β-mannanase on serum acute phase proteins ((**a**) AGP, and (**b**) HP)) and oxidative status markers ((**c**) SOD, and (**d**) GPx)) of weaning pigs.

Weaning and prolonged growth check can result in a status called oxidative stress, characterized by an excess of free radicals and insufficient protection by antioxidants [27]. Oxidative stress is directly linked with inflammation since oxidants are activators of the nuclear transcription factor NF-ĸB, the critical regulator of inflammation [28]. Previous studies have reported reduced oxidative stress and inflammation level in the weaning pig fed corn-SBM based diet by supplementation of NSP-degrading enzymes, such as xylanase and β-mannanase [29,30]. However, our result showed that the β-mannanase supplementation did not affect the concentrations of AGP and HP (APP parameters) as well as SOD and GPx (oxidative stress parameters; Figure 1). The discrepancies might be derived from the different platforms used for analysis. Previous studies mentioned above used mucosal surface as a marker of oxidative stress and feed induced immune response (FIIR), whereas the current results of oxidative stress and immune response were analyzed from the serum. Tiwari et al. [29] explained that the direct linkage between the mucosal surface and the intestinal digesta enables the detection of the quick changes in intestinal oxidative damage by nutritional interventions. Therefore, we suggest that further assessment of the β-mannanase effect on FIIR might show a promising result if applied by mucosal surface.

### 3.4. Intestinal Morphology

In this study, β-mannanase supplementation did not influence VH, CD and VCR in the ileum. However, the pigs fed the β-mannanase-supplemented diet had higher VH and VCR (*p* < 0.05) and lower CD (*p* < 0.05) in the jejunum compared with those fed the CON diet (Table 4). The VCR is a useful criterion for estimating the digestive capacity in the small intestine, and the NSP content in the fiber is one of the factors influencing the morphology of epithelial cells and the turnover rate of the gastrointestinal mucosa [31]. When mixed with water, the sNSP increased the viscosity of digesta whereas iNSP decreased the passage rate, providing a substrate that is slowly degraded by the cecal microbiota, resulting in the modulation of gut morphology [32]. Several investigations reported improved intestinal morphology (increased VC and VCR in the jejunum) by decreasing the level of sNSP in the weaning diet [33,34], mainly due to the reduced viscosity of digesta [35]. Therefore, it could be inferred that inclusion of β-mannanase in the weaning pigs’ diet might be attributed to reduced viscosity of digest by degrading β-mannan, which is one of the major sNSP in the diet, and thus it ameliorated the structural damage of the absorptive architecture.

### 3.5. Intestinal Microflora

There was a tendency to reduce the count of *E. coli* in the cecum (*p* = 0.08) in the pigs fed the β-mannanase-supplemented diet compared with those fed the CON diet (Table 5) even though there was no effect observed in ileum. It has been documented that the MOS in the large intestine act as a substrate for bacterial fermentation that produce short-chain fatty acids (SCFA) and may alter the intestinal microflora [36]. In human study, MOS could reduce the count of pathogenic bacteria (*Salmonella* and *E. coli*) in the intestine by the prevention of bacterial adhesion to the gut due to the saturation of mannose-specific binding site on their surface [37]. Previous study conducted with weaning pigs reported that applications of NSP-degrading enzymes such as mixtures of glucanase and xylanase significantly altered the production of SCFA and the profiles of gut-associated microflora in weaning pigs [38]. Similarly, Kim et al. [39] reported that the inclusion of multi-enzymes in the weaning diet modulated ileum and cecum microflora by increasing the count of *Lactobacillus spp*. and decreasing the count of *E. coli*. In the current study, the β-mannanase supplementation showed the potential to improve intestinal microflora by reducing the count of *E. coli*, which could result from the effect of liberated MOS by degrading β-mannan.

## 4. Conclusions

The results in the current study indicated that dietary β-mannanase supplementation did not affect growth performance, immunological and oxidative stress parameters, whereas it improved fat digestibility, intestinal integrity and microflora. This could be explained by the β-mannan (one of sNSP) hydrolyzing ability of β-mannanase, that could result in increasing fat digestibility, VH and VCR and reducing the count of *E. coli*.

## Figures and Tables

**Table 1:** Experimental diet formulation and chemical composition of the basal diets (as-fed basis) ^1^.

Ingredients, %	Phase 1	Phase 2
	(Day 0–14 Post-Weaning)	(Day 14–35 Post-Weaning)
Corn	17.8	38.22
Soybean meal, 44 % CP	22.08	24
HP300 ^2^	10	9
Whey powder	10	0
Lactose	15	5
Barley, dehulled	20	20
Soybean oil	1	1
Monocalcium phosphate	1.01	1.28
Limestone	0.92	0.92
L-Lysine·HCl	0.13	0.14
Vitamin premix ^3^	0.12	0.12
Trace mineral premix ^4^	0.12	0.12
Salt	0.1	0.1
Choline-Cl (25%)	0.1	0.1
Zinc oxide (ZnO) ^5^	0.1	0.1
Calculated chemical composition		
Metabolizable energy, kcal/kg	3400	3350
Crude protein, %	21.5	22.5
SID ^6^ lysine, %	1.35	1.23
SID methionine, %	0.39	0.36
Total Ca, %	0.8	0.7
STTD ^6^ P, %	0.4	0.35
Total NSP ^7^, %	10.93	12.89
Insoluble NSP ^7^, %	9.94	11.43
β-mannan ^8^, %	0.57	0.61

^1^ 0.06% β-mannanase (Hemicell^®^-HT: endo-1,4-β-mannanase from *Paenibacillus lentus*; Elanco Animal Health, Greenfield, IN, USA) replaced the same amount of corn in all Phase 1 and 2 diets for the β-mannanase treatment. The activity of β-mannanase was a minimum of 160 × 10^6^ Units (U)/kg product, resulting in 96,000 U/kg diet. ^2^ HP300 (Hamlet protein, Horsens, Denmark). ^3^ Supplied per kg diet: vitamin A, 8000 IU; vitamin D3, 1600 IU; vitamin E, 32 IU; vitamin B12, 12 g; vitamin K, 2.4 mg; D-biotin, 64 g; riboflavin, 3.2 mg; calcium pantothenic acid, 8 mg; niacin,16 mg. ^4^ Supplied per kg diet: Se, 0.1 mg; I, 0.3 mg; Mn, 24.8 mg; Cu·SO4, 54.1 mg; Fe, 127.3 mg; Zn, 84.7 mg; Co, 0.3 mg. ^5^ Zinc Oxide contained 3000 mg/kg. ^6^ SID = standardized ileal digestible, STTD = standardized total tract digestible. ^7^ Total non-starch polysaccharides (NSP) was calculated based on Pluske et al. [13], and insoluble NSP (iNSP) was calculated based on Jarowski and Stein, [14]. ^8^ β-mannan content in the diet was calculated based on Hsiao et al. [3] and Dierick [4].

**Table 2:** Effects of dietary supplementation of β-mannanase on growth performance of weaning pigs ^1^.

Items	Treatment ^2^		SEM ^3^	*p*-Value
	CON	β-Mannanase		
Body weight, kg				
day 0 post-weaning (initial)	6.96	6.95	0.430	0.98
day 14 post-weaning	9.18	9.37	0.601	0.82
day 35 post-weaning	16.79	17.01	1.078	0.89
Average daily gain, g/day				
day 0–14 post-weaning (Phase 1)	158	173	15.8	0.52
day 14–35 post-weaning (Phase 2)	354	364	26.4	0.80
day 0–35 post-weaning (Overall)	256	269	20.2	0.66
Average daily feed intake, g/day				
day 0–14 post-weaning (Phase 1)	273	295	19.0	0.43
day 14–35 post-weaning (Phase 2)	742	836	52.4	0.23
day 0–35 post-weaning (Overall)	558	537	35.2	0.31
Gain to feed ratio				
day 0–14 post-weaning (Phase 1)	0.557	0.585	0.019	0.31
day 14–35 post-weaning (Phase 2)	0.481	0.436	0.022	0.17
day 0–35 post-weaning (Overall)	0.519	0.510	0.015	0.69

^1^ Values represent means of eight replicates (pens) per treatment. ^2^ Dietary treatments: CON = basal diet, β-mannanase = basal diet + 0.06% β-mannanase. ^3^ Standard error of the means.

**Table 3:** Effects of dietary supplementation of β-mannanase on apparent total tract digestibility and nitrogen retention in weaning pigs ^1^.

Items	Treatment ^2^		SEM ^3^	*p*-Value
	CON	β-Mannanase		
Apparent total tract digestibility (%)				
Dry matter	87.92	87.78	1.148	0.93
Crude protein	86.63	86.56	1.591	0.98
Crude ash	56.14	58.30	4.371	0.74
Crude fat	70.13	76.51	4.783	0.01
Nitrogen (N) retention (g)				
N intake	6.79	7.05	0.058	0.65
Fecal N	0.91	0.95	0.109	0.81
Urinary N	1.77	1.86	0.263	0.81
N retention ^4^	4.11	4.24	0.293	0.77

^1^ Values represent means of five pigs per treatment (average BW 10.17 ± 1.35 kg). ^2^ Dietary treatments: CON = basal diet, β-mannanase = basal diet + 0.06% β-mannanase. ^3^ Standard error of the means. ^4^ N retention = N intake (g) − Fecal N (g) − Urinary N (g).

**Table 4:** Effects of dietary supplementation of β-mannanase on intestinal morphology of weaning pigs ^1^.

Items	Treatment ^2^		SEM ^3^	*p*-Value
	CON	β-Mannanase		
Jejunum				
Villus height (µm)	369.92	428.75	15.707	0.01
Crypt depth (µm)	244.67	212.50	11.182	0.05
Villus:Crypt ratio	1.58	2.03	0.104	0.01
Ileum				
Villus height (µm)	340.42	378.08	17.499	0.14
Crypt depth (µm)	188.83	191.58	10.862	0.86
Villus:Crypt ratio	1.83	2.08	0.141	0.23

^1^ Values represent means of five pigs per treatment (average anatomized BW: 17.17 ± 0.47 kg) slaughtered at d 35 post-weaning. ^2^ Dietary treatments: CON = basal diet, β-mannanase = basal diet + 0.06% β-mannanase. ^3^ Standard error of the means.

**Table 5:** Effects of dietary supplementation of β-mannanase on the count of *E. coil* in ileum and cecum of weaning pigs ^1^.

Items	Treatment ^2^		SEM ^3^	*p*-Value
	CON	β-Mannanase		
*E. coil* counts (log_10_ CFU/g fresh digesta)				
Ileum	7.40	6.68	0.509	0.33
Cecum	8.78	7.32	0.508	0.08

^1^ Values represent means of five pigs per treatment (average anatomized BW: 17.17 ± 0.47 kg) slaughtered at d 35 post-weaning. ^2^ Dietary treatments: CON = basal diet, β-mannanase = basal diet +0.06% β-mannanase. ^3^ Standard error of the means.
